# *Caulis Spatholobi* Ameliorates Obesity through Activating Brown Adipose Tissue and Modulating the Composition of Gut Microbiota

**DOI:** 10.3390/ijms20205150

**Published:** 2019-10-17

**Authors:** Chuanhai Zhang, Junyu Liu, Xiaoyun He, Yao Sheng, Cui Yang, Haoyu Li, Jia Xu, Wentao Xu, Kunlun Huang

**Affiliations:** 1 Beijing Advanced Innovation Center for Food Nutrition and Human Health, College of Food Science and Nutritional Engineering, China Agricultural University, Beijing 100083, China; zhangchuanhai.68@163.com (C.Z.); liujunyu0306@163.com (J.L.); raininghe@163.com (X.H.); shengyao314@163.com (Y.S.); 15549406754@163.com (C.Y.); kglihaoyu@sina.com (H.L.); xujia1995012@163.com (J.X.); xuwentao@cau.edu.cn (W.X.); 2 Key Laboratory of Safety Assessment of Genetically Modifed Organism (Food Safety), Ministry of Agriculture, Beijing 100083, China

**Keywords:** brown adipose tissue, *Caulis Spatholobi*, obesity, energy expenditure, gut microbiota

## Abstract

Obesity is associated with disrupted energy homeostasis and intestinal dysbiosis. *Caulis Spatholobi*, traditional Chinese medicine for herbal therapy, contains a wide range of bioactive compounds and has a specific pharmacological function. However, its effects on obesity and related metabolic disorder have remained largely unexplored. In this study, we showed that the water extract of Caulis Spatholobi (WECS) has a significant effect in inhibiting body weight gain, decreasing adiposity, maintaining glucose homeostasis, reducing insulin resistance and improving hepatic steatosis in diet-introduced obesity (DIO) mice. Besides, the administration of WECS significantly increased the expression levels of genes involved in the brown adipose tissue (BAT) activation and thermogenesis in DIO mice. Also, the activation of BAT treated with WECS was also confirmed in BAT primary cells. Mechanisms, the improvement of glucose homeostasis and insulin resistance may be related to the upregulated MAPK and AMPK pathways in white adipose tissue (WAT) and BAT. Notably, WECS also improved the obesity-induced gut microbiota dysbiosis, which induced an increase of anti-obesity and anti-diabetes related bacteria genus. In conclusion, *Caulis Spatholobi* can ameliorate obesity through activating brown adipose tissue and modulating the composition of gut microbiota. Our findings provide a novel perspective on Chinese medicine applications and provide a promising therapeutic approach for the treatment of obesity and metabolic disorders.

## 1. Introduction

Currently, the prevalence of obesity has become one of the most severe threats to human health [1]. Obesity is a result of excessive accumulation of body fat, which can cause many complications, such as diabetes, cardiovascular, and cerebrovascular diseases [2]. One of the leading reasons for obesity is that the energy intake exceeds the energy expenditure, and the excess energy accumulatively stores in white adipose tissue (WAT) as triglycerides (TGs) [3]. The traditional way to counteract obesity is to limit energy intake and implement lifestyle modifications. If the effects of this strategy are not satisfactory, long-term lifestyle modifications, perhaps under professional guidance, might be required. However, a more effective strategy to lose weight is to increase energy expenditure [4].

Mammalian adipose tissue can mainly divide into two types: WAT and brown adipose tissue (BAT) [5]. WAT is the largest repository of energy, whereas BAT is well known for its ability to burn energy. BAT is an essential tissue in newborn mammals, where non-shivering thermogenesis (NST) dominates to resist the cold environment [6,7]. Several studies have shown that adult human also has functional BAT [8,9], which was considered to be a very promising potential target for the treatment of obesity and its related metabolic diseases. The function and differentiation of BAT are affected by various factors, such as secretory proteins, transcription factors, and microRNAs [10,11,12]. Besides, appropriate stimulatory conditions also can enhance the expression of uncoupling protein 1 (UCP1) in subcutaneous WAT (sWAT) to generate beige cells, which has similar features to brown adipocytes, contain many small lipid droplets and have an elevated efficiency of oxidative phosphorylation. The central role of beige fat is to participate in adaptive thermogenesis, thereby increasing the energy expenditure of the body. Therefore, activation of BAT and induction of beige formation are effective ways to prevent and ameliorate obesity by increasing the energy expenditure.

Furthermore, increasing studies report that the gut microbiota is associated with obesity and the development of metabolic disorders [13]. The gut microbiota is composed of trillions of bacteria that contribute to nutrient acquisition and energy regulation. An increased ratio of Firmicutes/Bacteroidetes and changes in certain bacterial species can promote the development of obesity [14]. Other studies in obese animals suggest that the Clostridium cluster (Bacteroides spp., Oscillibacter spp., etc.) is related to fatty acid metabolism and gut permeability of the host [15]. Therefore, improving the gut microbiota is a promising strategy to ameliorate obesity. 

Traditional Chinese medicine applications and/or herbal therapy have a long history in Asian countries, dating back several thousands of years. As herbal medicine, Caulis Spatholobi, the vine stem of Spatholobus Erectus Dunn of the family Leguminosae, is specified in the Chinese Pharmacopoeia (2015 version) under the name of Ji-Xue-Teng; it contains a wide range of bioactive compounds [16]. Caulis Spatholobi has been used for centuries to promote health and longevity. Previous studies have shown that Caulis Spatholobi has hematopoietic, antiviral, antioxidant, antitumor, anti-inflammatory and sedative-hypnotic functions, and it influences the bidirectional regulation of tyrosine phosphorylation [17,18,19,20]. However, whether Caulis Spatholobi has any effect on anti-obesity or obesity-related disorders remains unknown.

In the current study, for the first time, we demonstrate that the water extract of Caulis Spatholobi (WECS) ameliorates obesity and improves obesity-induced metabolic disorders. Our results indicate that the herbal therapy with WECS is a practical approach to improve adiposity and its related metabolic diseases in diet-induced obesity (DIO) mice, mainly because of the activation of BAT, the induction of beiging in sWAT, and possibly the modulation of the composition of the gut microbiota. Our findings provide a novel potential therapeutic approach for the treatment of obesity and related metabolic disorders.

## 2. Results

### 2.1. WECS Ameliorates Obesity in DIO Mice

**Figure 1:**
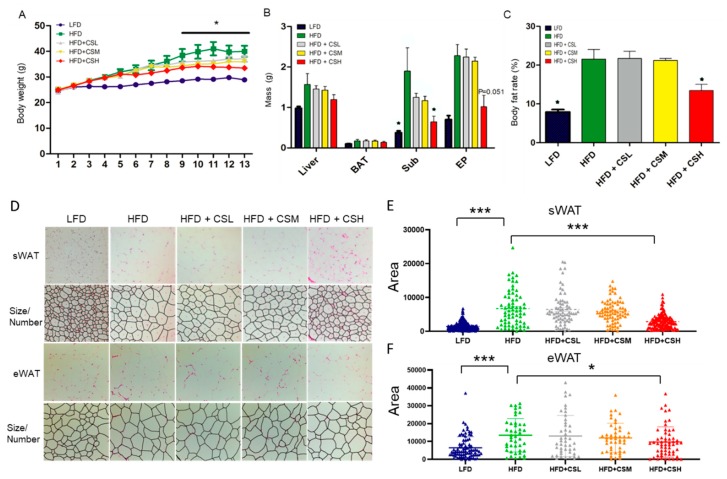
WECS treatment inhibits obesity in the DIO mice. The effect of different concentrations of WECS on body weight (**A**), organ weight (**B**), body fat rate (**C**), and the morphological characteristics of sWAT and eWAT (**D**). The cell area frequency of sWAT and eWAT in D are shown for the five different groups (Figure 1**E**,**F**). Bars represent the mean ± SEM, n = 7–9. * *p* < 0.05, *** *p* < 0.001, HFD+CSH compared with HFD control after 9 weeks (**A**).

To investigate the effect of WECS on anti-obesity, we developed the HFD induced obese mice and treated with WECS during the HFD process, and with low-fat diet (LFD) mice as normal control. In the HFD group, we treated the mice with different concentrations of WECS (Figure 1A). As a result, the administration of a high dose of WECS (HFD+CSH group, 1% m/m WECS solution) significantly decreased body weight gain in DIO mice (Figure 1A). Besides, WECS treatment significantly reduced the fat organ weight (Figure 1B) and body fat rate (Figure 1C) and restrained the sWAT and eWAT hypertrophy as measured by cell area (Figure 1D–F) compared with HFD control mice. These results imply that WECS can effectively inhibit body weight gain and fat accumulation in DIO mice. 

### 2.2. WECS Augments Whole-Body Energy Metabolism

**Figure 2:**
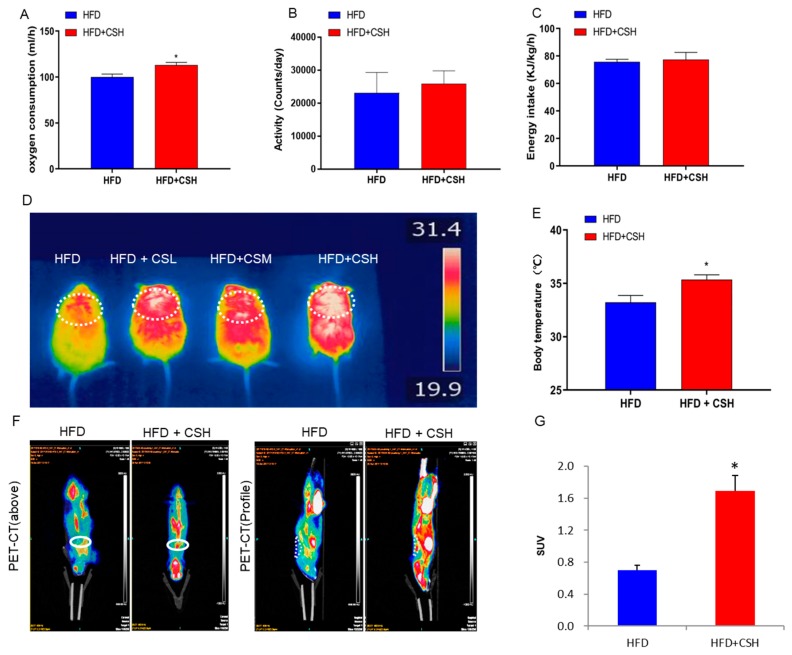
WECS augments whole-body energy metabolism. After 6 weeks of HFD and 7 weeks of treatment, WECS upregulated oxygen consumption (**A**). However, WECS has no effect on energy intake in mice (**B**) and activity (**C**). WECS treatment increased the thermal infrared signal (**D**) and rectal temperature (**E**) after cold stimulation of mice. PET-CT results show that WECS increased the BAT activity in mice (**F**). The bar graph represents the average uptake of 18F-FDG in BAT (**G**). Bars represent the mean + SEM, *n* = 3–5. * *p* < 0.05 compared with HFD control.

Given only the obesity-related phenotype of mice in the HFD+CSH group was significantly different from that in the HFD control group, we studied only these two groups further. To further explore the effects of WECS on the energy metabolism in mice, we performed the respiratory metabolic system test and found that WECS significantly increased the oxygen consumption in DIO mice (Figure 2A). However, there was no significant difference in the activity and energy intake between the HFD+CSH and HFD groups mice (Figure 2B,C). These results strongly indicated that WECS enhanced whole-body energy metabolism due to the increased oxygen consumption in DIO mice. Besides, WECS treatment significantly increased the thermogenesis of interscapular location, which is same as the interscapular BAT location, and rectal temperature after cold stimulation in WECS treatment groups compared with the HFD control group (Figure 2D,E). Consistently, the positron emission tomography-computed tomographic (PET-CT) analysis shows that WECS treatment dramatically increased the positive PET signal of BAT, which represents the glucose utilization, in HFD+CSH mice compared with HFD control mice (Figure 2F,G). These results suggested that WECS enhanced energy metabolism may due to the increased activation of BAT.

### 2.3. WECS Induces BAT Activity and Beige Generation in sWAT

**Figure 3:**
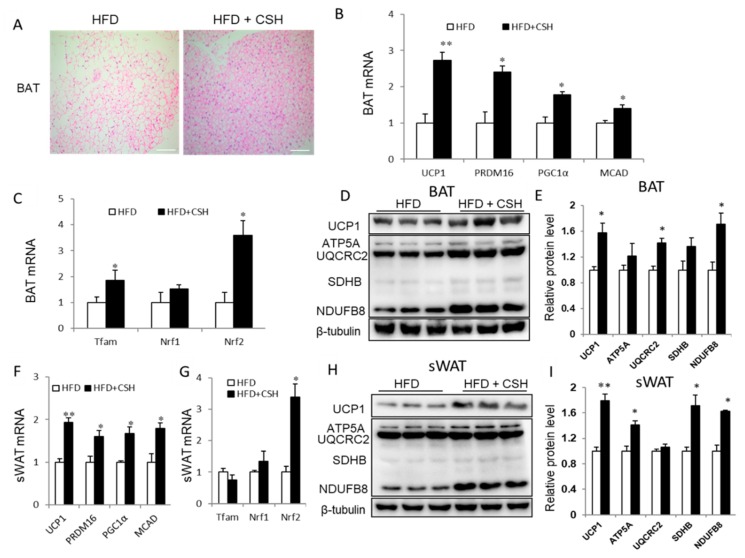
WECS activates BAT and induces browning of SWAT. Histology analysis showed that WECS treatment decreased the lipid droplets in the BAT(**A**). Scale bars, 100 μm. Besides, WECS treatment upregulated the expression of thermogenic genes and some mitochondrial genesis genes in BAT (**B** and **C**) and sWAT (**F** and **G**) in HFD mice. Besides, WECS upregulated the expression of UCP1 and some proteins related to oxidative phosphorylation in BAT (**D**) and sWAT (**H**). Relative protein levels of UCP1 and OXPHOS in BAT and sWAT (**E** and **I**). Bars represent the mean + SEM, *n* = 3–5. * *p* < 0.05, ** *p* < 0.01 compared with HFD control.

Based on the above findings, we surmised that WECS could contribute to the activation of BAT and the induction of beiging effects in sWAT. We observed that the morphological features of BAT and sWAT show a denser cell structure in the HFD+CSH group compared with the HFD control group (Figure 3A and Figure 1D). Indeed, the mRNA levels of thermogenic genes of BAT in mice, including UCP1, Prdm16, PGC1a, and MCAD were dramatically increased in BAT from WECS-treated (HFD+CSH) mice (Figure 3B). The expression levels of mitochondrial biogenic transcription factors, including Tfam and NRF2, were markedly increased in BAT from HFD+CSH mice (Figure 3C). We further confirmed that the mitochondrial oxidative phosphorylation (OXPHOS, including ATP5A, UQCRC2, SDHB, and NDUFB8) and expression of UCP1 increased in BAT from HFD+CSH mice at the protein level (Figure 3D,E). Consistently, the PET-CT analysis shows that the positive PET signal is increased in BAT from HFD+CSH mice (Figure 2F,G).

Similar to BAT, beige cells are also rich in mitochondria for adequate energy consumption. We investigated the expression of several thermogenic and mitochondrial genes in sWAT. The results show that both thermogenic genes (UCP1, Prdm16, PGC1a, and MCAD) and mitochondrial gene NRF2 expression levels were significantly increased in sWAT from HFD+CSH mice (Figure 3F,G). Notably, there was also a significant increase in UCP1 protein levels (Figure 3H,I). Furthermore, the expression of OXPHOS proteins, including ATP5A, UQCRC2, SDHB, and NDUFB8, was significantly upregulated in sWAT from HFD+CSH mice (Figure 3H,I). Besides, we also performed the analysis of related molecular phenotypes in eWAT. Interestingly, there was no difference both in thermogenic genes expression and/or OXPHOS, and UCP1 protein levels in eWAT treated with or without WECS (Supplementary Figure S1A,B). This may due to the treatment concentration of WECS dosage not meet the conditions for eWAT to produce beige cells. These results indicate that WECS significantly increased BAT activity and induced the generation of beige adipocytes in sWAT.

**Figure 4:**
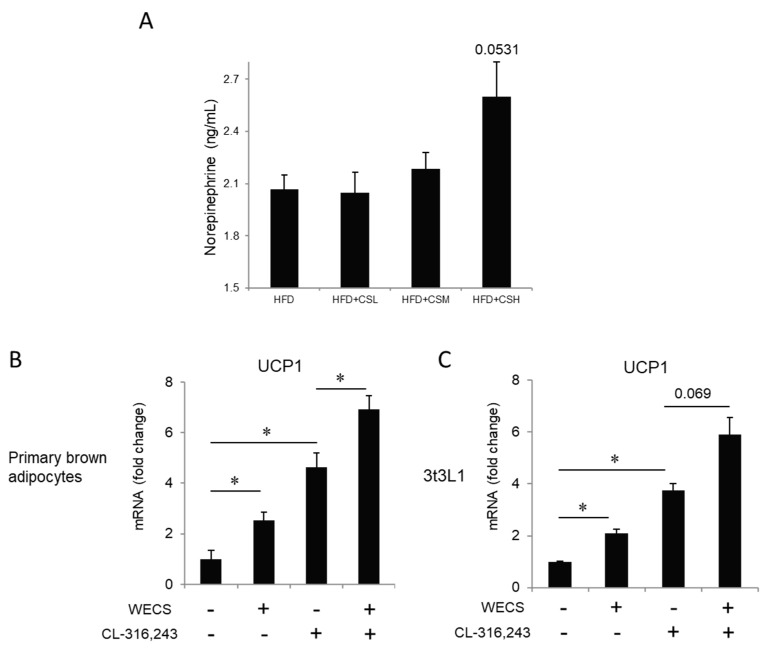
The effect of WECS on the sympathetic pathway. The concentration of norepinephrine in serum (**A**). The effect of WECS and β3 adrenergic agonist (CL-316,243) in brown adipocytes and 3T3L1 after browning induction (**B** and **C**). Bars represent the mean + SEM, *n* = 3–5. *p < 0.05.

As we knew, the activity of BAT is closely related to the sympathetic nervous system (SNS) [21,22]. Therefore, we measured whether WECS activates sympathetic nerves by examining the concentration of norepinephrine (NE) in the serum by enzyme-linked immunosorbent assay. The results show that the average NE concentration was higher after WECS treatment, at the margin of statistical significance (*p* = 0.0531) (Figure 4A). This finding indicates that WECS plays a particular role in activating the SNS. Besides, we checked the effects of WECS on UCP1 expression levels in brown primary adipocytes and 3T3L1 cells induced by brown adipogenic induction cocktail after β3 adrenergic receptor agonist (CL-316,243) stimulation (Figure 4B,C). The β3 adrenergic receptor agonist (CL-316,243) is widely used to mimic cold stimulation. The results indicate that WECS also exerts a positive effect on UCP1 expression in brown adipocytes and beige adipocytes (*p* = 0.069) with β3 adrenergic receptor agonist stimulation.

### 2.4. WECS Increases Mitochondrial Biogenesis And Oxygen Consumption

**Figure 5:**
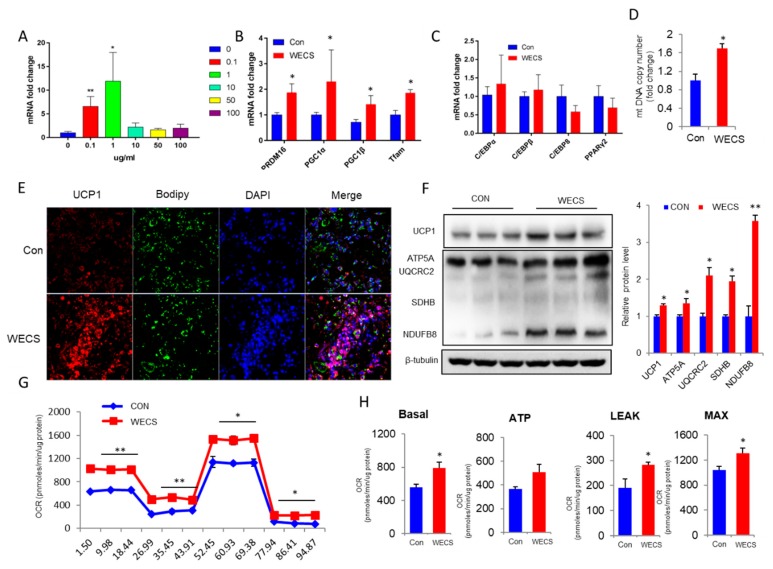
WECS increases mitochondrial biogenesis and oxygen consumption. Dose-dependent effects of WECS on UCP1 expression in brown adipocytes (**A**). Thermogenic and mitochondrial biogenic gene expression in brown adipocytes treated with 1 μg/mL WECS or DMSO (**B**). The expression levels of adipogenic marker genes (**C**). The mitochondrial copy numbers (**D**). Representative images of UCP1 staining (**E**). The protein levels of UCP1 and OXPHOS in brown adipocyte (F). Brown adipose primary cells were treated with 1 μg/mL WECS or DMSO during 6 days of brown adipogenesis; OCR at day 6 (**G**). The OCR-related basal metabolic rate, ATP levels, maximum oxygen consumption, and proton leakage, were all significantly increased (**H**). Bars represent the mean + SEM, *n* = 3–5. * *p* < 0.05, ** *p* < 0.01 compared with the HFD control.

To further confirm the direct effect of WECS on brown adipocytes, we isolated and used brown primary adipocytes in mice to perform an in vitro brown adipocytes differentiation assay, treated with/without WECS. WECS increased the expression of UCP1 in a dose-dependent manner with the maximum effect at a concentration of 1 μg/mL (Figure 5A). As a result, UCP1 levels increased dramatically in the WECS-treated (CS) group compared with the solvent control group, as measured by quantification of the protein expression by Western blot and immunostaining (Figure 5E,F). Furthermore, the expression levels of OXPHOS proteins, including ATP5A, UQCRC2, SDHB, and NDUFB8, were significantly upregulated in the CS group (Figure 5F). Moreover, WECS treatment also increased the expression levels of other BAT-related thermogenic genes, such as PRDM16 and PGC1α (Figure 5B), although no significant effect on the expression of adipogenic genes, including c/ebpα, c/ebpβ, c/ebpδ, and pparγ, was observed (Figure 5C). Indeed, the expression levels of key genes in mitochondrial biogenesis, such as Tfam, were significantly increased by WECS treatment in the CS group compared with the control group (Figure 5B). We also observed that WECS dramatically augmented the mtDNA copy number (Figure 5D). At the same time, oxygen consumption was significantly increased by WECS treatment and was further boosted by the addition of carbonyl cyanide 4-(trifluoromethoxy) phenylhydrazone, a potent mitochondrial oxidative phosphorylation uncoupler (Figure 5G). Correspondingly, the oxygen consumption rate (OCR)-related basal metabolic rate, ATP levels, maximum oxygen consumption, and proton leakage were all significantly increased (Figure 5H). Taken together, these results indicate that WECS markedly increased the mitochondrial biosynthesis and cellular respiration of brown adipocytes.

### 2.5. WECS Improves Glucose Homeostasis and Insulin Resistance

**Figure 6:**
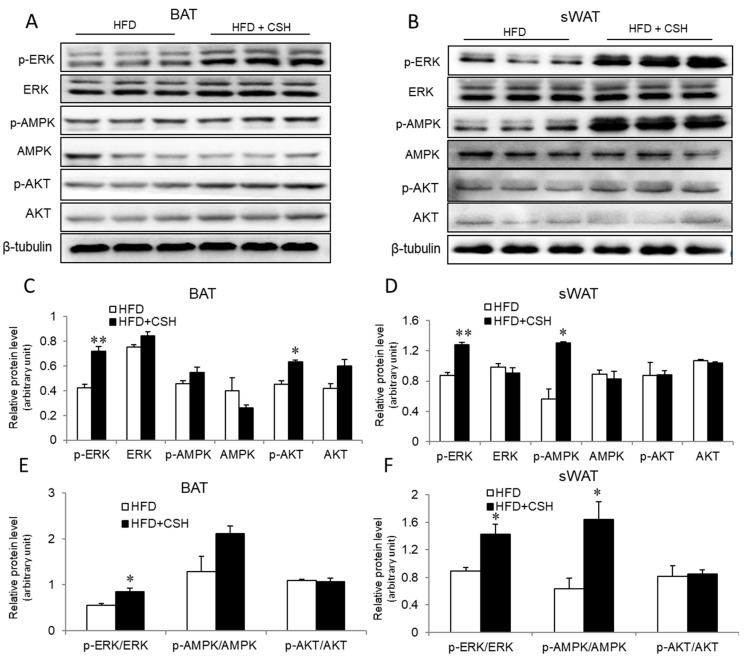
The expression levels of proteins involved in insulin signaling were analyzed after WECS treatment. The phosphorylation of MAPK was significantly increased after the WECS treatment in BAT and sWAT (**A**,**B**). The relative protein level of A and B were shown in (**C**,**D**). The relative protein level of p-ERK/ERK, p-AMPK/AMPK, p-AKT/AKT were showed in (**E**,**F**). Bars represent the mean + SEM, n = 3. *p < 0.05, **p < 0.01 compared with the HFD control group.

Adipose tissues are known to play an essential regulatory role in glucose homeostasis and insulin sensitivity. Given the improved morphological and molecular phenotypes of BAT and sWAT, we hypothesized that WECS can improve glucose homeostasis. To this end, we performed the glucose tolerance test (GTT) and insulin tolerance test (ITT). The results show that WECS significantly improved the glucose tolerance and insulin sensitivity of DIO mice (Supplementary Figure S2A–D). As we knew, several proteins such as ERK, AMPK, and AKT, are associated with the insulin signaling pathway. Therefore, we next analyzed the insulin signaling pathway by immunoblotting. Notably, we found that WECS treatment can increase the expression levels of phosphorylation of ERK(P-ERK)/ERK in BAT ((Figure 6A,C,E), sWAT (Figure 6B,D,F) and eWAT (Supplementary Figure S1C,D). Interestingly, the phosphorylation of AMPK was significantly increased only in sWAT (Figure 6B,D,F). These results suggest that the WECS improves glucose homeostasis and insulin resistance, at least in part, through ERK and AMPK pathways.

### 2.6. WECS Relieves Hepatic Steatosis

**Figure 7:**
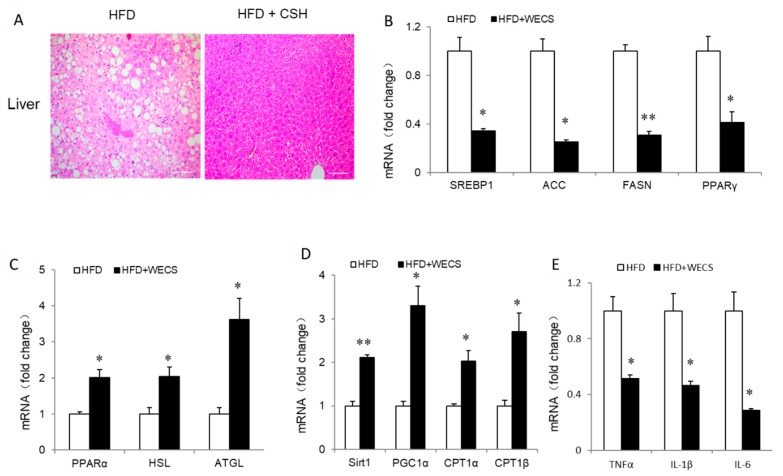
WECS relieves hepatic steatosis. Hematoxylin and eosin staining from liver sections in control HFD mice or HFD mice treated with WECS (**A**). Scale bars, 100um. The real-time PCR analysis of lipid synthesis (**B**), lipid lipolysis (C), fatty acid oxidation related gene (**D**), and inflammatory factor-related gene (**E**) expression. Bars represent the mean + SEM, *n* = 3. * *p* < 0.05, ** *p* < 0.01 compared with the HFD control group.

Obesity usually causes hepatic steatosis. To investigate the beneficial effect of WECS on hepatic steatosis, we firstly observed the lipid droplets within the liver from DIO mice treated with/without WECS. As a result, WECS treatment dramatically decreased the lipid droplets amount within the liver (Figure 7A). Accordingly, WECS treatment obviously downregulated the expression of lipid synthesis related gene, including SREBP1, ACC, FASN, and PPARγ, in the DIO mouse liver (Figure 7B), while the expression of lipid lysis related genes(PPARα, HSL, and ATGL), and fatty acid oxidation (FAO) genes(SIRT1, PGC1α, CPT1α, and CPT1β), dramatically increased (Figure 7C,D). Besides, the inflammatory genes, including TNF-α, IL-6, and IL-1β, were significantly decreased after WECS treatment in the liver (Figure 7E). Furthermore, the serum analysis shows that WECS treatment reduced TG and low-density lipoprotein levels while increasing HDL levels of the serum in HFD mice (Supplementary Figure S3A). Interestingly, WECS treatment significantly reduced the LDH (lactate dehydrogenase) levels, which suggests that WECS may exert some protective effects on the liver (Supplementary Figure S3B). In summary, these results demonstrated that WECS treatment could effectively ameliorate the hepatic steatosis in DIO mice.

### 2.7. WECS Modulated the Composition of the Gut Microbiota

**Figure 8:**
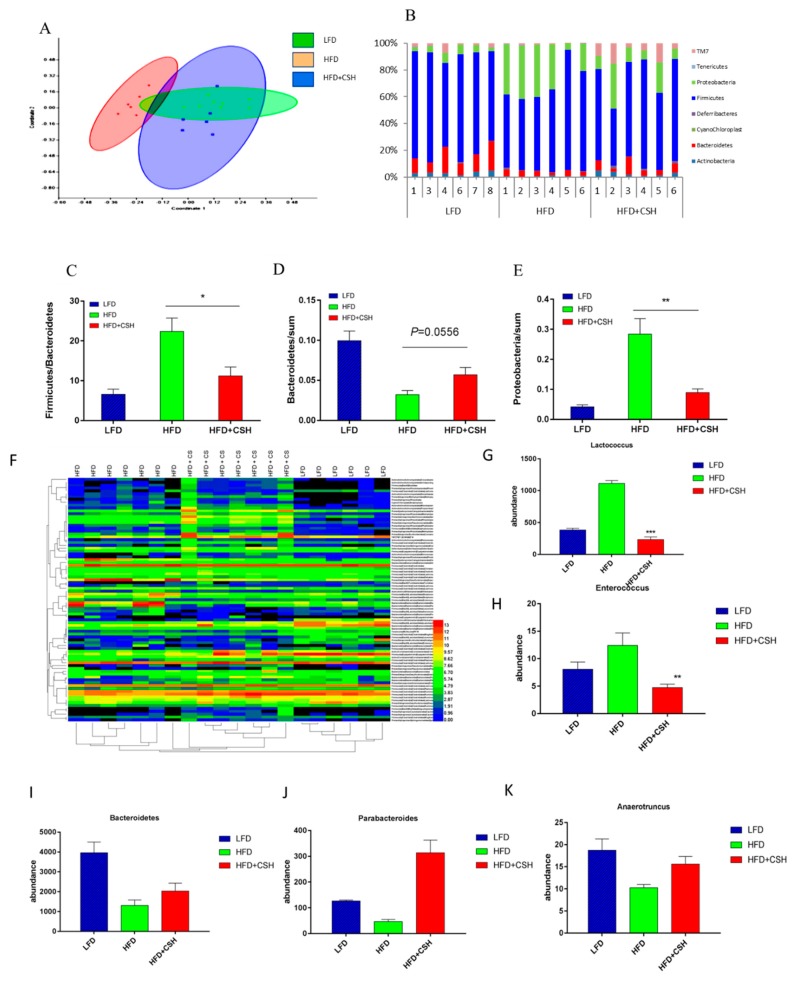
WECS modulates the composition of the gut microbiota. Microbiota composition in feces of HFD mice treated with or without 1% WECS and LFD mice were analyzed using next-generation sequencing (*n* = 7 for each group). NMDS and Multivar/One-way ANOSIM analysis were used to explore the similarity between groups (**A**). Results of the bacterial taxonomic profiling at the phylum level of intestinal bacteria from different mouse groups (**B**–**E**). The heatmap shows that the abundance of 85 OTUs was significantly altered in HFD-fed mice (**F**). We also screened several representative microbes associated with obesity (**G**–**K**). Bars represent the mean + SEM, *n* = 6–7. * *p* < 0.05, **p < 0.01, ***p < 0.01 compared with the HFD control.

It was well known that gut microbiota is associated with obesity and the development of metabolic disorders. We examined the effects of WECS on gut microbiota composition by performing a pyrosequencing-based analysis of bacterial 16S rRNA (V3–V5 region) in caecal feces. Non-metric MDS (NMDS) analysis revealed distinct clustering of microbiota composition (Figure 8A). Furthermore, taxonomic profiling demonstrated that treatment with 1% WECS reduced the ratio of Firmicutes to Bacteroidetes and the amount of bacteria from the Proteobacteria phylum in DIO mice similar to those found in LFD mice (Figure 8B–E).

To identify the specific bacterial phylotypes, which were altered by HFD feeding (HFD) and HFD feeding with WECS treatment (HFD+CSH), we analyzed the changes of operational taxonomic units (OTUs) in each group (Figure 8F). Compared with LFD mice, HFD feeding altered 85 OTUs, which including 50 increased OTUs and 35 decreased OTUs. However, WECS treatment reduced or even reversed the changing trends of 46 of these OTUs. Detailed analysis of the 46 OTUs indicated that changes in *Enterococcus spp.* and *Lactococcus lactis* (which were found to be increased by HFD and to be positively correlated with obesity in previous studies) were all reversed by WECS treatment (Figure 8D,E). Moreover, during the HFD, WECS treatment increased a variety of bacterial species that were negatively correlated with obesity, including *Parabacteroides*, *Bacteroidetes*, *Anaerotruncus* and *Bifidobacterium* (Figure 8G–K). We also examined these 46 OTUs one by one. We selected a total number of 19 OTUs that were significantly changed by WECS (Table 1). These results show that WECS modulated the gut microbiota of DIO mice, which, as a result, more closely resembled the microbiota composition of health LFD mice. Collectively, our findings indicated that WECS treatment might play a role in promoting the anti-obesity effect though improving the composition of the gut microbiota in obese mice.

## 3. Discussion

Like many traditional Chinese medicines, *Caulis Spatholobi* has been used to promote health and longevity for centuries. Because most Chinese medicines need to be decocted in water, many studies research water extracts of Chinese medicine [15,23,24]. *Caulis Spatholobi* was found to promote hematopoiesis and showed antiviral activity in vivo or in vitro [17,25]. However, the effects of this traditional Chinese medicine on the gut microbiota, metabolic metabolism in adipose tissue and obesity had not been investigated. In this study, we confirmed that WECS could effectively prevent diet-induced obesity by enhancing BAT activity and modulating the composition of the gut microbiota.

We first confirmed the weight reduction effect of WECS in vivo. Considering the food nibbling behavior of mice might affect the recording of food intake, we applied the respiratory metabolism system to measure the amount of food consumed in mice in the current study. During our 13-week experiments, we observed that WECS treatment reduced the body weight and WAT accumulation in DIO mice, without influencing the food or energy intake. Obesity is also associated with many metabolic syndromes. For example, obesity often leads to glucose metabolism disorder and insulin resistance [26]. MAPK and AMPK are classic pathways that regulate glucose metabolism [27]. WECS treatment improved the glucose tolerance and increased the expression of related MAPK and AMPK pathway proteins in BAT and WAT of DIO mice. We speculate that WECS activates the p-ERK/ERK, p-AMPK/AMPK, and p-AKT/AKT pathways and thereby regulates glucose metabolism.

Obesity caused by an energy imbalance; the energy intake exceeds the energy expenditure [28]. Our experiments confirmed that WECS treatment increased energy expenditure by increasing the oxygen consumption and physical activity of DIO mice. These phenomena illustrate that WECS increased the catabolism of DIO mice. BAT is essential for maintaining the body temperature of rodents and infants by thermogenesis [29,30]. Once BAT is activated, calorie uptake in BAT is even higher than that in muscle, liver, and WAT [30]. BAT-dependent NST based on the uncoupling of mitochondria, which is achieved by UCP1, an essential BAT marker [31,32,33]. In our study, WECS significantly increased the expression of UCP1 at both the mRNA and the protein level. Additionally, the mitochondrial function has an impact on whole-body metabolism that is closely related to BAT function [6]. Tfam and NRF1 regulate mitochondrial DNA replication and biogenesis [34], and OXPHOS is related to mitochondrial oxidative phosphorylation [35]. In the present study, WECS increased the expression of mitochondrial genes and OXPHOS in BAT of DIO mice. It indicated that WECS enhanced the function of BAT mitochondria.

As is well known, intestinal microflora is also closely related to obesity [13]. In this study, we found that WECS not only reduced the bodyweight of DIO mice but also modulated the intestinal microflora composition (Figure 6). In general, HFD feeding led to a marked change in the categories of intestinal microflora in mice, whereas WECS treatment tended to reverse this trend. The *Bacteroidetes phylum* was suggested to be mainly responsible for protection against increased adiposity [36], while a high ratio of *Firmicutes* to *Bacteroidetes* correlated to increased body weight [37]. In our study, WECS increased the abundance of the *Bacteroidetes phylum* and decreased the ratio of *Firmicutes* to *Bacteroidetes* in mice. Proteobacteria, positively correlated with obesity, was also significantly reduced by the WECS treatment in DIO mice. Previous studies have shown that *Enterococcus spp.* can decrease the total antioxidant capacity of the colon content [38,39]. In the present study, WECS treatment lowered the abundance of *Enterococcus* to a number that was even lower than that of LFD mice. WECS increased the abundance of microorganisms that are good for losing weight, such as *Bifidobacterium* [14,39] and *Lactococcus lactis* [40], and decreased the abundance of microorganisms that are bad for losing weight, like *Parabacteroides* and *Anaerotruncus* [15]. These findings suggest that WECS also may contribute to weight loss by improving the composition of gut microbiota. 

**Figure 9:**
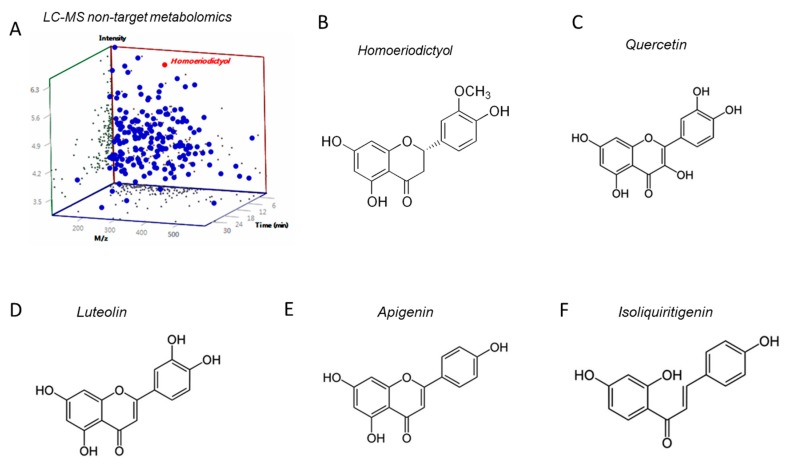
The LC-MS non-target metabolomics of WECS (**A**). We screened out a compound- homoeriodictyol which is the most abundant isoflavone in WECS. The molecular structural formula of homoeriodictyol (**B**) and serval flavonoids with a similar structure of Homoeriodictyol, such as Quercetin(**C**), Luteolin(**D**), Apigenin(**E**), Isoliquiritigenin (**F**).

However, the specific causal relationship between obesity and the WECS-induced improvement of gut microbiota remains uncertain. More comprehensive research is needed to confirm the exact relationship between WECS and intestinal flora and its therapeutic effects on obesity. Besides, WECS is a mixture of various compounds, and in this study, it has not confirmed which molecule is responsible for the main pharmacological action. However, we performed LC-MS non-target metabolomics and screened a high-proportioned compound-*homoeriodictyol*, which belongs to one of the isoflavones (Figure 9A,B). The chemical structure of *homoeriodictyol* is very similar to several other flavonoids, which imply that they may have similar functional effects (Figure 9C–F). The main functional components in WECS, are isoflavones and catechin. Considering that several studies have pointed out that isoflavones [41,42] and catechin [43,44] are associated with obesity and intestinal flora, we should continue to pay attention to isoflavones and catechin in future research. Individually, we will pay more attention to *homoeriodictyol*, which screened out form LC-MS analysis of WECS in the future study.

Collectively, the current study, for the first time, confirmed that WECS has an anti-obesity effect. Our data proved that Caulis Spatholobi plays an effective role in inhibiting obesity in DIO mice through the activation of BAT, induction of beiging in sWAT, and modulation of the composition of the gut microbiota. Caulis Spatholobi may be a promising novel candidate for Chinese medicine applications to ameliorate obesity and related metabolic diseases, including diabetes. Predictably, our study provides a new perspective on herbal therapy for the treatment of obesity and metabolic disorders.

## 4. Materials and Methods

### 4.1. Animals

All experimental procedures were conducted and the animals were used according to the Guide for the Care and Use of Laboratory Animals published by the U.S. National Institute of Health and approved by the Animal Ethics Committee of China Agricultural University, Beijing (the approval ID of this study is KY1700019, authorization date is April 18, 2017). Male C57BL/6J mice (15–20 g, 4 weeks old) were purchased from Vital River Laboratories (Beijing, China) and housed (four animals/cage) under 22 ± 2 °C and 55% ± 10% humidity with 12 h light/12 h dark cycle in a specific pathogen-free animal room. Animals were acclimated to the environment for 5 days with normal commercial basic diet and sterile water ad libitum before the experiment.

The DIO model was generated as follows: eight mice were fed an LFD, and the rest were fed an HFD until the weight difference was over 20%. We marked the mice fed an LFD and sterile water as the vehicle control group (LFD, *n* = 8). The HFD mice were divided into four groups: negative control group (HFD, *n* = 8) fed an HFD and sterile water, and three treatment groups fed an HFD and WECS solutions (HFD+CSL, HFD+CSM, HFD+CSH with 0.25%, 0.5%, 1% m/m WECS solution for drinking, respectively, *n* = 8). The experiment lasted for another 13 weeks.

The LFD contained fat that provided 10% of the total calories, and the HFD contained fat that provided 60% of the total calories (Supplementary Table S1). We prepared WECS solutions at the specified concentrations, and after 20 min of ultrasonic oscillation, the solution was clarified and used as drinking water for the mice. All animal diets and WECS powder were kept at 4 °C before use. We prepared the WECS solutions every 2 days before use. 

WECS (50:1, from Yunnan Province of China) was purchased from Nanjing DASF Biotechnology Co. Ltd. We performed non-target LC-MS mass spectrometry analysis on this product. The specific component data can be found in supplementary materials.

### 4.2. Glucose Tolerance Test and Insulin Tolerance Test

At the last week of the experiment, we performed the GTT on the mice. After overnight fasting for 16 h, animals were administered 1.5 g/kg BW glucose solution, which was dissolved in normal saline, via intraperitoneal injection. We determined blood glucose concentrations from the tail vein with a blood glucose meter (ACCU-CHEK, Shanghai, China) at 0, 15, 30, 60, 90 and 120 min. For the insulin tolerance test (ITT), the mice fasted for 4 h (9:00 AM to 1:00 PM) and the mice were administered human insulin (0.7 U/kg Humulin R; Novo Nordisk) by intraperitoneal injection. Blood glucose concentrations were determined from the tail vein with blood glucose meter (ACCU-CHEK) at 0, 15, 30, 45 and 60 min after the insulin injection.

### 4.3. Rectal Temperature and Energy Expenditure

During the last week of the experiment, the mice were placed in a cold room (4 °C) for 4 h. We evaluated the cold-induced thermogenesis by measuring the rectal temperature with a temperature sensor (AT210, Zhongyidapeng, Shenzhen, China). Then we took photos of the animals by a handheld infrared camera (FLIR T600) on a whiteboard.

### 4.4. Metabolic Rate and Physical Activity

Oxygen consumption and physical activity were determined at the 12th week of the experiment with a TSE lab master (TSE Systems, Germany). Mice were acclimated to the system for 20–24 h, and then we measured oxygen consumption (VO_2_ and VCO_2_) over the next 24 h. The animals were maintained at 24 °C in a 12-h light/dark cycle with free access to food and water. We measured the voluntary activity of each mouse using an optical beam technique (Opto-M3, Columbus Instruments, Columbus, OH, USA) over 24 h and expressed as 24 h average activity. Then, the respiratory exchange ratio (RER) was calculated.

### 4.5. Body Composition Measurements

The total fat and lean masses of mice after a 7-week treatment with either vehicle or WECS were assessed with the Small Animal Body Composition Analysis and Imaging System (MesoQMR23-060H-I, Nuimag Corp., Shanghai, China), according to the manufacturer’s instructions.

### 4.6. PET-CT Imaging

We conducted PET-CT imaging with the Siemens Inveon Dedicated PET (dPET) System and Inveon Multimodality (MM) System (CT-SPECT) (Siemens INVEON PET/CT, Germany) at the Peking Union Medical College Hospital. We injected 500 mCi 18F-fluorodeoxyglucose (18F-FDG) into the tail vein of the mice. The mice were fixed on the instrument during the measurement. After 60 min, we analyzed the images on the Inveon Acquisition Workplace. The absorption and utilization of glucose in different parts and organs were indicated by color changes in different parts of the mice.

### 4.7. Histology and BODIPY Staining

Tissues fixed with 4% paraformaldehyde were sectioned after embedment in paraffin. We prepared multiple sections for hematoxylin-eosin staining. We incubated cells grown on poly-L-lysine-pretreated coverslips (Sigma-Aldrich, St. Louis, MO, USA) in 5% goat serum for 1 h at room temperature after fixation with 1% formalin at room temperature for 1 h; then, they were incubated with BODIPY (Thermo Fisher Scientific, Waltham, MA, USA) for 2 h at room temperature. All images were acquired with the BX51 system (Olympus, Tokyo, Japan).

### 4.8. Gut Microbiota Analysis

The mice were separated, and one mouse was placed per cage, and the fresh feces (about 100 mg) were taken from the bottom of the cage and put into 2-mL aseptic centrifuge tubes with forceps sterilized by high pressure. Stool samples were snap-frozen in liquid nitrogen before storage at −80 °C. DNA was extracted using a fecal DNA isolation kit (FUDEAN, Beijing, China). The DNA was sent to Nuo he source bio Mdt InfoTech Ltd for metagenomic sequencing. We analyzed the raw sequencing data using the Oracle VM VirtualBox system. Then we investigated the OTUs for analysis of the overall change and diversity of the intestinal microbial community. Multivar/NMDS and Multivar/One-way ANOSIM analysis with PAST software were used to analyze the differences between samples. A heatmap was made with Heml Software (version 1.0.3.7).

### 4.9. Brown Adipocyte Differentiation

We took mouse BAT primary cells from neonatal mouse within 24 h after birth, BAT was cut and suspended in PBS, and a BAT single-cell suspension was obtained after filtration. These BAT cells were then cultured in basal medium (containing 80% DMEM, 20% fetal bovine serum, and 1% penicillin and streptomycin) until the cells had reached more than 90% confluency. Then, we treated the cells with a brown adipogenic induction cocktail (DMEM) containing 10% fetal bovine serum, 1% penicillin and streptomycin, 20 nM insulin, 1 mM dexamethasone, 0.5 mM isobutylmethylxanthine, 125 nM indomethacin, and 1 nM 3,3,5-triiodo-L-thyronine (T3) for the first 2 days. The medium was then replaced by the medium supplemented with only insulin and T3 every other day. The cells were treated with or without WECS (1 μg/mL) for 6 days during brown adipogenesis. BAT differentiation medium was used as the solvent, and 0.1 μg/mL, 1 μg/mL, 10 μg/mL, 50 μg/mL and 100 μg/mL solution were used as the material for the in vitro tests. At day 6, fully differentiated adipocytes were used for the experiments.

### 4.10. Measurements of Cellular Respiration

We treated mouse BAT primary cells both with and without WECS (1 μg/mL) for 6 days during brown adipogenesis. We measured O2 consumption of fully differentiated adipocytes at day 6 with an XF24-3 extracellular flux analyzer (Agilent Technologies, Santa Clara, CA, USA). Basal respiration was also assessed in untreated cells.

### 4.11. Quantitative Real-Time PCR

We isolated total RNA using a total tissue RNA isolation kit (ET101-01, TransGen Biotech, Beijing, China). We used equal amounts of RNA to synthesize cDNA with the transScript One-Step gDNA Removal and cDNA Synthesis SuperMix kit (AT311-03, TransGen Biotech, Beijing, China). We performed quantitative real-time PCR (qRT–PCR) was performed in triplicate using SYBR Green, 96-well plates and the Real-Time PCR System (Bio-Rad, Hercules, CA, USA). Each well was loaded with a total of 20 μL containing 2 μL of cDNA, 2 × 0.4 μL of target primers, 7.2 μL of water and 10 μl of SYBR Fast Master Mix. We performed hot-start PCR for 45 cycles, with each cycle consisting of denaturation for 5 s at 94 °C, annealing for 15 s at 58 °C and elongation for 10 s at 72 °C. We used the cyclophilin expression to normalize the mRNA expression. The primers used are shown in Supplementary Table S2.

### 4.12. Western Blot Analysis

We loaded an equal amount of cell lysate (20 µg per lane) into a 12% SDS-polyacrylamide gel after denaturation with SDS loading buffer. After electrophoresis, we transferred proteins to a PVDF membrane and incubated with blocking buffer (5% fat-free milk) for 1 h at room temperature. The following antibodies were added overnight: anti-UCP1 (Abcam, ab10983, 1:1000 diluted in 5% BSA, 0.0025% Tween-20 in 1× TBS solution), anti-Oxphos (Abcam, ab110413, 1:1000 dilution), anti-MAPK (CST, #8960S, 1:1000 dilution), anti-pMAPK (CST, #9102S, 1:1000 dilution), anti-AKT (CST, #9272S, 1:1000 dilution), anti-pAKT (CST, #9271S, 1:1000 dilution), anti-AMPK (CST, #2532S, 1:1000 dilution), anti-pAMPK (CST, #2535S, 1:1000 dilution), anti-β-actin (CST, #8457, 1:1000 dilution) and anti-β-tubulin (CST, #2146, 1:1000 dilution). These primary antibodies were incubated overnight in a 4 °C refrigerator. The membrane was incubated with horseradish peroxidase-conjugated secondary antibodies for 1 h at room temperature. All signals were visualized and analyzed by Clinx ChemiCapture software (Clinx, Shanghai, China).

### 4.13. Immunofluorescence Staining

We stained differentiated cells with anti-human UCP1, followed by an Alexa 488-conjugated secondary antibody (Invitrogen, Carlsbad, CA, USA), BODIPY (Thermo Fischer Scientific, Waltham, MA, USA) and DAPI (Leagene, Beijing, China), complying with manufacturers’ the procedure. Brown adipocytes were positive for both UCP1 and BODIPY. We stained negative controls with the omission of a primary antibody. Images were taken by Zeiss laser scanning confocal microscopy (LSM780, Oberkochen, Germany).

### 4.14. Statistical Analysis

We used a single-factor analysis of variance (ANOVA) followed by a two-tailed Student’s t-test for comparisons. We presented almost all data as means ± SEM. Significant differences were considered when *p* < 0.05. Graph-Pad Prism 7 (GraphPad Software, San Diego, CA, USA) was used for data analysis.

## Figures and Tables

**Table 1:** OTU group trends after WECS treatment of HFD-fed mice.

	OTU that Changed by the Water Extract of *Caulis Spatholobi*	Increase/Decrease Of The HFD+CSH Group Compared with the HFD Group	*p*-Value
Level of family	Clostridiaceae	↑	0.036487607
	Lachnospiraceae	↓	<0.0001
	Ruminococcaceae	↑	0.033080246
	Erysipelotrichaceae	↑	0.047428491
Level of genus	*Facklamia*	↓	0.0142127
	*Enterococcus*	↓	0.0035
	*Lactobacillus*	↑	0.002309373
	*Candidatus Arthromitus*	↑	0.028112884
	*Ruminococcus*	↑	0.036487607
	*Bacteroides*	↓	<0.0001
	*Prevotella*	↓	<0.0001
	*Adlercreutzia*	↑	0.010054934
	*Dehalobacterium*	↓	0.011452914
	*Lactococcus*	↓	<0.0001
	*Oscillospira*	↑	0.005455646
	*Leuconostoc*	↓	<0.0001
	*Anaerotruncus*	↑	0.0159
	*Roseburia*	↓	0.029076172
	*Parabacteroides*	↑	0.0018

↑ Up arrow represents up-regulation; ↓ Down arrow represents down-regulation.

## Supplementary Materials

Supplementary materials can be found at https://www.mdpi.com/1422-0067/20/20/5150/s1.
