# Supplementary material for: Caulis Spatholobi Ameliorates Obesity through Activating Brown Adipose Tissue and Modulating the Composition of Gut Microbiota
Source: Int J Mol Sci. 2019 Oct 17;20(20):5150. doi: 10.3390/ijms20205150 (PMC6829277; doi:10.3390/ijms20205150)
Supplement: Supplementary file 1 [file ijms-20-05150-s001.zip › Supplementary/supplementary-tablesú¿updatedú⌐.docx]

**Table S1.** The feed ingredients.

|  | **LFD** | | |  | **HFD** | | |
| --- | --- | --- | --- | --- | --- | --- | --- |
|  | **Percentage m/m** |  | **Percentage of total energy** |  | **Percentage m/m** |  | **Percentage of total energy** |
| Protein | 19.2 |  | 20 |  | 26 |  | 20 |
| Carbohydrate | 67.3 |  | 70 |  | 26 |  | 20 |
| Fat | 4.3 |  | 10 |  | 35 |  | 60 |
| total: |  |  | 100 |  |  |  | 100 |
| kcal/g | 3.85 |  |  |  | 5.24 |  |  |

**Table S2.** Primer sequences.

| Primer | Forward | Reverse |
| --- | --- | --- |
| CEBP/α | GCGGGAACGCAACAACATC | GTCACTGGTCAACTCCAGCAC |
| CEBP/β | TGACGCAACACACGTGTAACTG | AACAACCCCGCAGGAACAT |
| CEBP/δ | CGACTTCAGCGCCTACATTGA | GAAGAGGTCGGCGAAGAGTT |
| Cideα | TGCTCTTCTGTATCGCCCAGT | GCCGTGTTAAGGAATCTGCTG |
| HSL | CTGAGATTGAGGTGCTGTCG | CAAGGGAGGTGAGATGGTAAC |
| ATGL | GCGCCAGGACTGGAAAGAAT | TGAGAACGCTGAGGCTTTGAT |
| MCAD | ACTCGAAAGCGGCTCACAA | ACGGGGATAATCTCCTCTCTGG |
| SREBP1 | GGAGGGGTAGGGCCAACGGCCT | CATGTCTTCGAAAGTGCAATCC |
| FASN | AGCTGCCAGAGTCGGAGAAC | TGTAGCCCACGAGTGTCTCG |
| ACC | GCTGCTCGGATCACTAGTGAA | TTCTGCTATCAGTCTGTCCAG |
| NRF1 | CAACAGGGAAGAAACGGAAA | GCACCACATTCTCCAAAGGT |
| NRF2 | TAGATGACCATGAGTCGCTTGC | GCCAAACTTGCTCCATGTCC |
| PGC1α | ACAGCTTTCTGGGTGGATT | TGAGGACCGCTAGCAAGTTT |
| PGC1β | CGTATTTGAGGACAGCAGCA | TACTGGGTGGGCTCTGGTAG |
| PPARγ2 | TCGCTGATGCACTGCCTATG | GAGAGGTCCACAGAGCTGATT |
| PRDM16 | GAAGTCACAGGAGGACACGG | CTCGCTCCTCAACACACCTC |
| Cyclophilin | CAAATGCTGGACCAAACACAA | GCCATCCAGCCATTCAGTCT |
| SIRT1 | GCCAGAGTCCAAGTTTAGAAGA | CCATCAGTCCCAAATCCAG |
| TNFα | TGGGCCTCTCATGCACCACC | GAGGCAACCTGACCACTCTCCCT |
| IL6 | AGACAAAGCCAGAGTCCTTCAG | GCCACTCCTTCTGTGACTCCAG |
| IL1β | TTCAGGCAGGCAGTATCACTC | GAAGGTCCACGGGAAAGACAC |
| Tfam | GTCCATAGGCACCGTATTGC | CCCATGCTGGAAAAACACTT |
| UCP1 | GGCAAAAACAGAAGGATTGC | TAAGCCGGCTGAGATCTTGT |
| β-globin | GAAGCGATTCTAGGGAGCAG | GGAGCAGCGATTCTGAGTAG |
