# Supplementary material for: Caulis Spatholobi Ameliorates Obesity through Activating Brown Adipose Tissue and Modulating the Composition of Gut Microbiota
Source: Int J Mol Sci. 2019 Oct 17;20(20):5150. doi: 10.3390/ijms20205150 (PMC6829277; doi:10.3390/ijms20205150)
Supplement: Supplementary file 1 [file ijms-20-05150-s001.zip › Supplementary/Supplementary.docx]

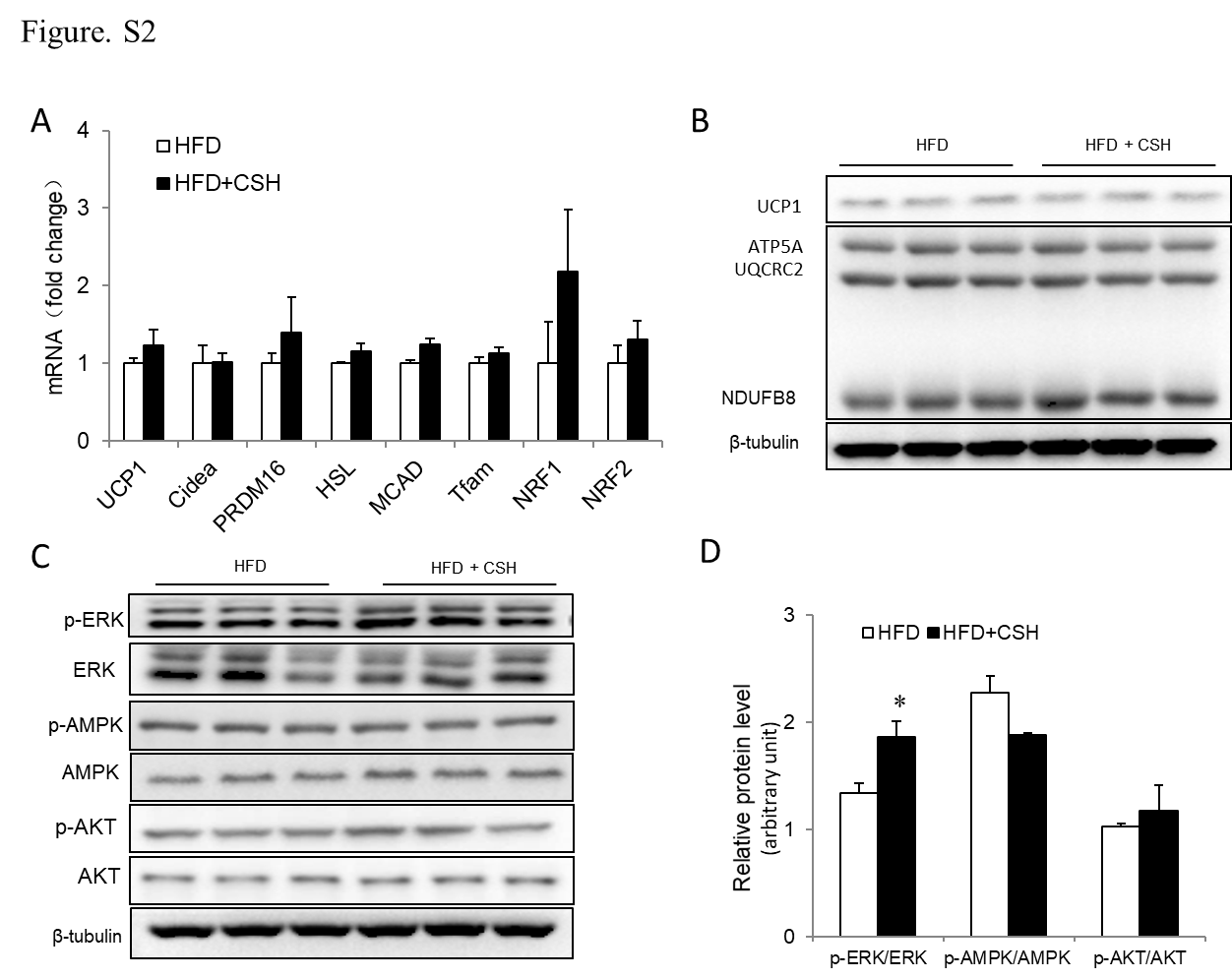


**Supplementary figure S1.** The thermogenic genes and mitochondrial genes were analyzed after WECS treatment (A and B). The phosphorylation of ERK was significantly increased after the WECS treatment in eWAT(C). The relative protein level of p-ERK/ERK, p-AMPK/AMPK,p-AKT/AKT were showed in D. Bars represent the mean + SEM, n = 8. **P* < 0.05, ***P* < 0.01 compared with the HFD control group.

**
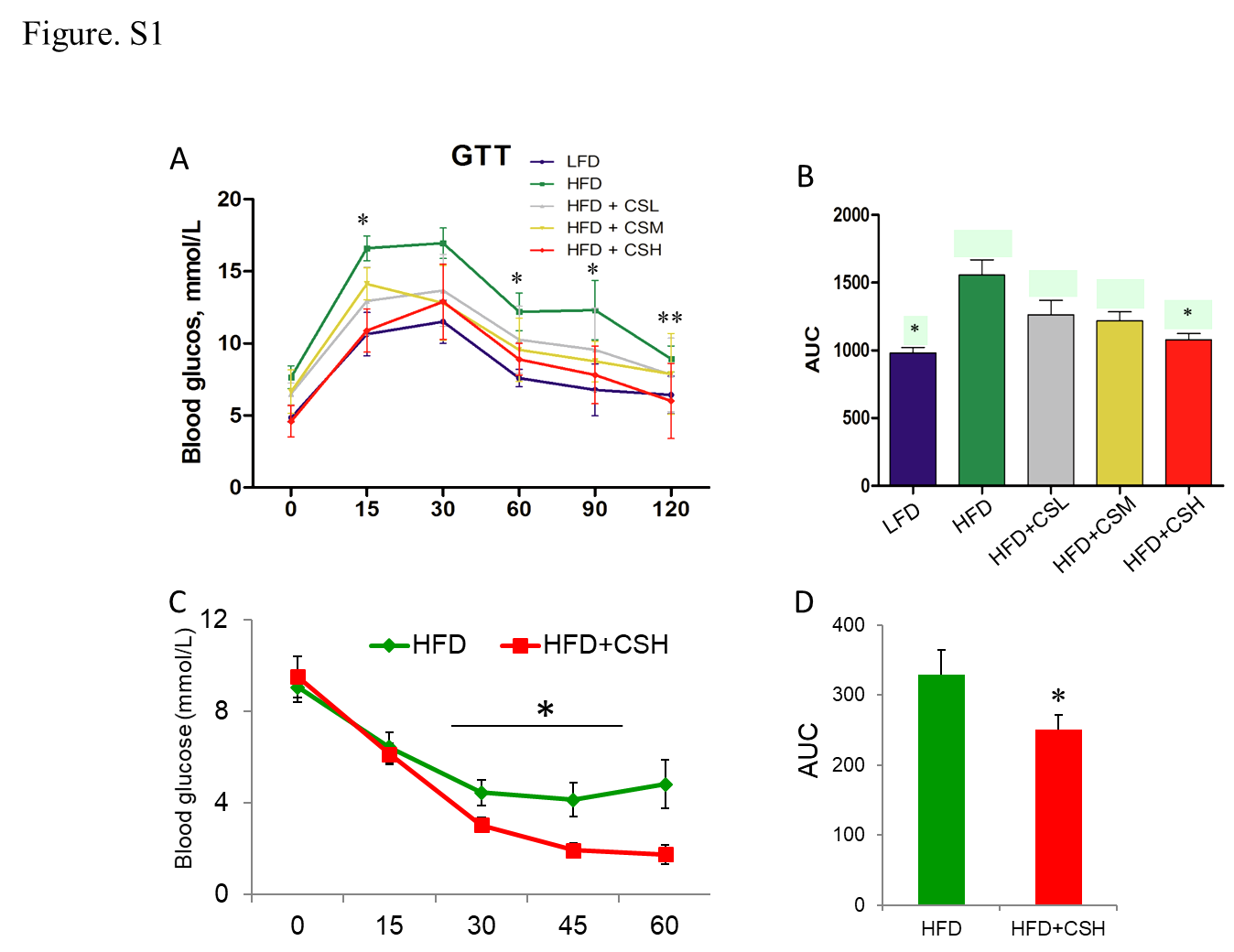
 Supplementary figure S2.** WECS treatment improves the glucose tolerance and insulin sensitivity of HFD-fed mice (A and B). The area under the curve also confirms this result (C and D). Bars represent the mean + SEM, n = 8. **P < 0.05,* ***P < 0.01* compared with the HFD control group.


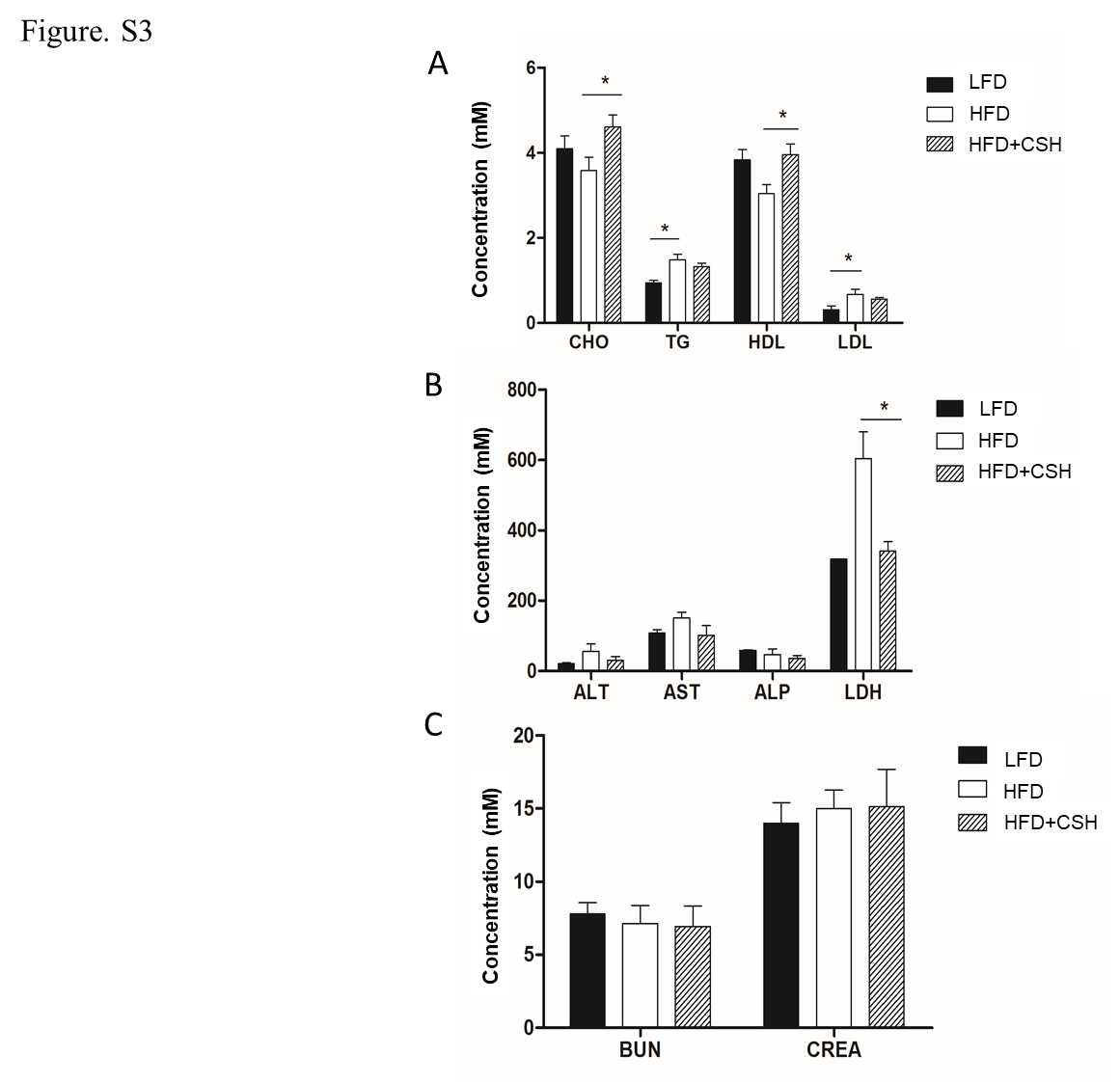


**Supplementary figure S3.** WECS lowers the blood lipid levels (A) and protects the liver (B). Bars represent the mean + SEM, n = 8. **P* < 0.05 compared with the HFD control group.
